# TOPSAN: a collaborative annotation environment for structural genomics

**DOI:** 10.1186/1471-2105-11-426

**Published:** 2010-08-17

**Authors:** Dana Weekes, S Sri Krishna, Constantina Bakolitsa, Ian A Wilson, Adam Godzik, John Wooley

**Affiliations:** 1 Joint Center for Structural Genomics, Bioinformatics Core, Sanford-Burnham Medical Research Institute, 10901 N. Torrey Pines Road, La Jolla, CA 92037, USA; 2 Joint Center for Structural Genomics, The Scripps Research Institute, 10550 N. Torrey Pines Road, La Jolla, CA 92037, USA; 3 Joint Center for Structural Genomics, Bioinformatics Core, Center for Research in Biological Systems, University of California, San Diego, 9500 Gilman Drive, La Jolla, CA 92093, USA; 4 Joint Center for Molecular Modeling, Sanford-Burnham Medical Research Institute, 10901 N. Torrey Pines Road, La Jolla, CA 92037, USA

## Abstract

**Background:**

Many protein structures determined in high-throughput structural genomics centers, despite their significant novelty and importance, are available only as PDB depositions and are not accompanied by a peer-reviewed manuscript. Because of this they are not accessible by the standard tools of literature searches, remaining underutilized by the broad biological community.

**Results:**

To address this issue we have developed TOPSAN, The Open Protein Structure Annotation Network, a web-based platform that combines the openness of the wiki model with the quality control of scientific communication. TOPSAN enables research collaborations and scientific dialogue among globally distributed participants, the results of which are reviewed by experts and eventually validated by peer review. The immediate goal of TOPSAN is to harness the combined experience, knowledge, and data from such collaborations in order to enhance the impact of the astonishing number and diversity of structures being determined by structural genomics centers and high-throughput structural biology.

**Conclusions:**

TOPSAN combines features of automated annotation databases and formal, peer-reviewed scientific research literature, providing an ideal vehicle to bridge a gap between rapidly accumulating data from high-throughput technologies and a much slower pace for its analysis and integration with other, relevant research.

## Background

Structural biology uses experimental methods, such as X-ray crystallography and NMR spectroscopy, to provide atomic-level information about the three-dimensional shapes of biological macromolecules. Such detailed information often delivers a critical component of the puzzle that has to be solved in order to understand the function of a macromolecule. Information provided by structural biology, while essential, by itself is not enough to decipher protein function without associated information from biochemistry, molecular and cellular biology, genomics, and other fields of biology. Historically, experimental structure determination of a protein was a long and painstaking process, which was generally initiated only after a significant body of biochemical and biological evidence about the function of a specific protein had been assembled. The very length of the structure determination process, with many steps extending into months, offered ample time to perform additional analyses, integrate all available information, and even carry out additional experiments to clarify the most interesting questions that were raised during the investigation. Such a multi-pronged approach to the characterization and analysis of a structure of a single protein was typically conducted by single investigator groups with broad experience or through collaboration among multiple laboratories with synergistic expertise. These collaborations were forged by standard mechanisms of communication in the scientific community.

This standard model of a structural biology project is now changing, largely because of the development of technological platforms capable of high-throughput protein structure determination. These platforms have reduced the cost and shortened the time for determination of the structure of a novel protein [1]. A significant part of this development came from the formation of large, specialized production centers as part of the NIH/NIGMS Protein Structure Initiative and from the contributions of other similar centers around the world, which are collectively referred to as Structural Genomics (SG) centers. In the last few years, the U.S.-based SG centers alone have solved more than 3,000 non-redundant protein structures, and similar numbers of total structures have been deposited in the Protein Data Bank by the combined contributions of the Japanese and European SG centers. Proteins determined by SG groups include hundreds of examples of the first representative(s) of protein families and other structures of interest; indeed, many are directly relevant to human health. However, the success in advancing technical aspects of protein structure determination has created, rather unexpectedly, a crisis of sorts, as the pace of solving and releasing new structures proceeds independently of, and much faster than, any other complementary experimental work. Thus, given this vastly increased scale of new structures being determined, insufficient time is available for performing the additional biological and biochemical studies to arrive at a complete story for a given protein concurrent with the structure determination process. As a result, most SG-determined structures, despite their significant novelty and importance, are available only as PDB depositions and are not accompanied by a peer-reviewed manuscript. By not being described in ways that would make them accessible by the standard tools of literature searches, they remain underutilized by the broad biological community.

At the same time, the interest, relevant expertise, and resources to complete the characterization and analysis of the proteins determined by SG centers do exist in the broader community, i.e., mostly outside of the structure determination centers. However, the traditional mechanisms of forming collaborations and communicating results cannot keep pace with the high-throughput production of SG centers. Such traditional mechanisms include forming personal networks that arise from extended discussions and interactions at meetings and from local collegial interactions within universities and institutes. These mechanisms have become woefully inadequate as the period for protein structure determination has shrunk to days/weeks rather than months/years. Given the very diverse set of proteins that are worked on by PSI centers, the expertise needed to fully analyze each of them is difficult to find even in the largest labs. Finally, another important difference between an SG center and a standard structural biology lab is the high-throughput aspect of structural genomics. With one structure being determined, on average, each working day, there is simply not enough time to integrate relevant, non-structural data and/or to follow a traditional approach to establishing collaborations with appropriate groups with synergistic experience.

Structural biology is not the only field struggling to maintain a balance between high-throughput data accumulation and a much slower pace for its analysis and integration with other, relevant research. The former is driven by the rapid pace of technological development, whereas the latter is limited by the standard methods of data analysis and assimilation. The equivalent amount of time and effort that was once needed to sequence a single gene can now yield the sequence of an entire genome; similarly, the effort needed a few years ago to analyze the expression pattern for a single gene can now yield a genome-size DNA expression array. In contrast, the time needed to research a particular question and look for possible connections to other data and/or experiments, as well as the time to find and consult with a colleague who is knowledgeable in another, often connected field, are not easily changed by technology. As a result, a significant percentage of data obtained by high-throughput techniques remain suspended in the no-man's land of "unpublished results"; for instance, the almost 18,000 microarray experiments in the Stanford microarray database have led to only 449 publications [2], and almost 40% of fully sequenced bacterial genomes (and an increasing number of eukaryotic ones) did not lead to a single publication [3].

This growing gap between data creation and analysis has led to exploration of new approaches for exchanging and disseminating information, ranging, for example, from blogs to wikis to networking sites. Most of these recent attempts have been enabled by the emergence of the Internet, which has changed, and is still changing, the way people communicate, both in science and beyond. It is interesting to note that the most important developments in the Internet era, from the Internet itself to the concept of the World Wide Web [4], were driven by the needs of the scientific community to communicate and exchange complex information. Many other experiments in what we would now call community annotations were also driven by the technological developments in science. For instance, the rapid growth of genomic sequences driven by the technical developments in the field of DNA sequencing spurred the initiation of other novel approaches to deal with the relentless increase in data generation, such as genome annotation jamborees (e.g., Flybase [5]) and dedicated websites devoted to annotation and exchange of data on specific organisms, which became early models for social scientific networks and unstructured research data exchange. More recently, the emergence of the "Wikipedia" concept [6] has shown how much the creative use of relatively simple computer technologies can change the dynamics of information collection and dissemination and allow unprecedented collaborations between geographically dispersed communities of users. The success of Wikipedia has inspired the biological community to adopt this model to collect and disseminate scientific information [7]. However, Wikipedia focuses on the collection and dissemination of existing, already verified information, as exemplified in one of the fundamental rules of Wikipedia; namely, it includes "no original research" http://en.wikipedia.org/w/index.php?title=Wikipedia:No_original_research. On the other hand, creating new knowledge is a critical part of science. Wikipedia's dependence on already verified information allows it to avoid issues such as impartial mechanisms for ensuring the reliability of information (e.g., peer review) and assigning credit for individual contributions. These problems are addressed by peer-reviewed literature, but at the cost of slower speed of dissemination, as well as other constraints imposed by the inherently inefficient structure of the institutionalized peer-review process.

Most biology focussed wikis, such as WikiPathways [8], Proteopedia [9], WikiProteins [10], PDBWiki [11], Wikigenes [12], and GeneWiki [13], while adding novel extensions to visualize biological information, adhere largely to the Wikipedia model. Only some of them, such as WikiGenes, go beyond the standard Wikipedia model and address, or at least acknowledge, some of the issues important in creating new knowledge, such as authorship tracking. Here, we describe our attempt to develop a model of an open scientific collaboration platform that seeks to achieve a balance among the openness and ease of use of a wiki, the need for rigorous validation and for recognizing the importance or pride of authorship, the achievement of the value typical for peer-reviewed literature, and the speed and accuracy of a database. We have concentrated mostly on the needs of the emerging structural genomics field, which studies the vast unexplored sectors of the protein universe, but we are aware that the same issues are faced by other scientific fields, including higher throughput structural biology efforts, such as the recently initiated PSI:Biology http://www.nigms.nih.gov/Initiatives/PSI/psi_biology/. Mechanisms similar to those we outline here could be employed to address similar issues in these fields. By focusing on protein structures, TOPSAN has a particular relationship to the recently developed Proteopedia [9] or PDBWiki, which are wiki-style encyclopedias mainly devoted to the 3D structures of proteins. Despite a similar research field (structural biology), TOPSAN differs from both Proteopedia and PDBWiki by scope (concentrating on unannotated structures solved by structural genomics centers vs. all structures in the PDB) and overall goals (developing new knowledge vs. propagating and popularizing existing knowledge). The Proteopedia and PDBWiki pages on structures solved by structural genomics centers are mostly empty, as no published information exists on these structures.

TOPSAN, **T**he **O**pen **P**rotein **S**tructure **A**nnotation **N**etwork http://topsan.org, was developed at one of the PSI high-throughput production centers, the Joint Center for Structural Genomics (JCSG), and has since become a collaborative project involving personnel from other PSI centers, as well as from other institutions. Since its inception, the JCSG has recognized the need for high-throughput annotation and the further analysis of its targets for structure determination and, subsequently, for solved structures; these processes are conducted through a combination of automated annotations, detailed knowledge-based analyses, and follow-up collaborations to advance the functional understanding of the structures being determined. A particularly illustrative example that predates our current instantiation of TOPSAN can be seen as a result of our determination of a novel thymidylate synthase, TM0449 [14] from *Thermotoga maritima*. Previously known only from genomic complementation studies in *Dictyostelium *[15], the non-homologous, biochemical pathway of thymidylate synthesis emerged from relative obscurity with the structure determination of TM0449 by the JCSG and spawned a series of informative mechanistic studies that elucidated details of this pathway in a series of subsequent publications in peer-reviewed journals [16-18].

**Figure 1:**
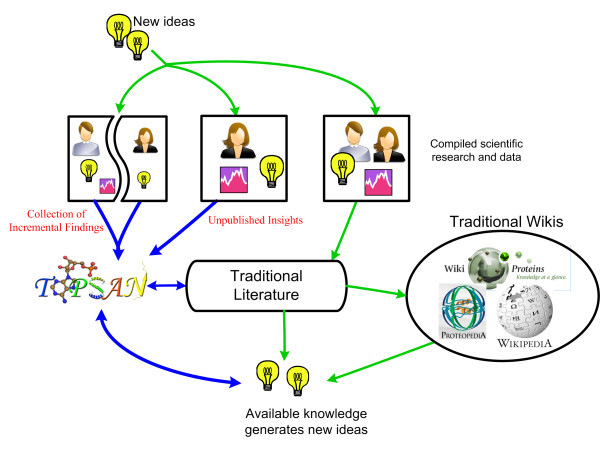
**Information flow**. Traditional flow of information (green) comes from an idea, or an empirical observation, (symbolized by a light bulb) that is developed and supported by subsequent scientific research and data (symbolized by a graph) and then submitted to a traditional peer-reviewed journal. If accepted, new ideas and hypotheses may be propagated from the published results and this process is iterated. Once published, the information is then considered knowledge, as opposed to an idea or an untested hypothesis, and would be accepted in the traditional wikis. However, insights about the data may be lost if the compiled data and research are not published in a timely manner. Even a very good idea or hypothesis may never be propagated if they are not assembled with associated data to make a publishable module required for traditional journals. TOPSAN information flow (blue) proposes to not only accept, but also encourage intellectual contributions ranging from speculation to hypotheses to extensive data collections/analyses. While a full, peer-reviewed publication remains an ultimate goal, at any given time most TOPSAN pages would represent prepublication stages. TOPSAN attempts to expand an iterative research process, typically restricted to a single research group, to a distributed, virtual collaboration.

In this article, we describe our ongoing implementation of TOPSAN [19] and lay out the capabilities of this specific platform for new potential users, so as to encourage the community to become part of this new model of open, collaborative science. An innovative feature of TOPSAN is its goal of serving as a collaborative network that focuses on creating new knowledge. We aim to achieve this goal by enabling instant collaborations among scientists, each with different expertise and often different perspectives. While anyone can read the database, participation in the system is open only to registered users, which allows for both authorship tracking, as well as for quality control. In contrast to Wikipedia and its scientific extensions, novel contributions are not only accepted, but are actively solicited and encouraged. **Figure **1 depicts the standard scientific information flow and the place occupied by TOPSAN, differentiating it not only from other wiki implementations, but also from traditional peer-reviewed literature and/or standard annotation databases.

Authorship tracking mechanisms ensure both proper assignment of credit and accountability of contributors. Entries are peer-reviewed by users with established records of accomplishment and credentials to ensure content reliability; at this point, the review is largely performed by senior JCSG scientists. All users of the system implicitly accept the collaborative rules, as any TOPSAN entry is, in fact, an open invitation to collaborate with the SG center that solved this particular structure. This invitation is supported by significant preliminary data and results, such as biological material (clones, purified proteins) and sequence and structure analyses that often provide detailed hypotheses on the function of the newly solved protein. Such collaborations can lead to traditional, peer-reviewed papers, and, encouragingly, many of the TOPSAN entries are, in fact, progressing this way.

One view of the TOPSAN system then is that of a stepping-stone to peer-reviewed publications, where the openness of our system allows for the immediate establishment of collaborations and TOPSAN pages are treated as a mechanism to link individual collaborators and unpublished results to achieve the ultimate goal of publishing in a, standard, peer-reviewed journal. Another, more far-reaching view of TOPSAN is that of a "live" protein annotation and collaboration platform, i.e., a platform that might lead to novel forms of ongoing, virtually continuous scientific communication and knowledge creation. For collaborative science, this approach offers a new paradigm especially pertinent to the "omics" era. While we personally favor this view, the actual outcome will ultimately be determined by the general community of users and contributors.

## Results and Methods

### TOPSAN and a process for annotating and understanding proteins

**Figure 2:**
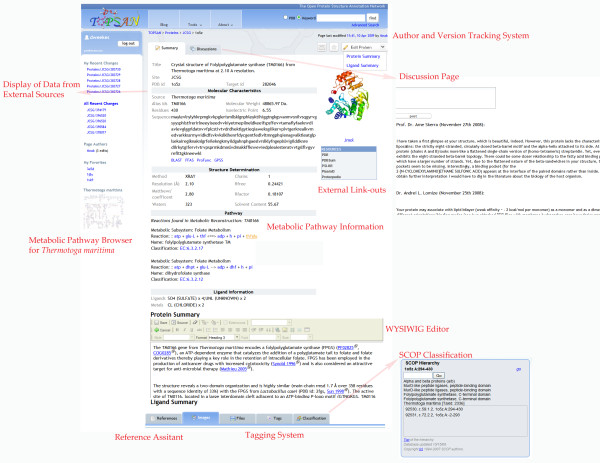
**A TOPSAN protein page, displaying information from the JCSG database, links to other important information sources, system to easily add references, classification widget with current SCOP annotations**[25], **discussion pages, WYSIWYG editor, author and version tracking system (critical for developing publication authorship), tagging system, metabolic pathway information (at this point for *Thermotoga maritima *proteins only)**.

TOPSAN http://www.topsan.org is a new type of communication platform for annotations and follow-up analysis and research on proteins. At this point, TOPSAN focuses on proteins targeted or solved by structural genomics centers; however, users are free to create pages on any protein they are interested in, and many, actually most, of the TOPSAN pages are compiled for proteins whose structures are not yet determined. Furthermore, many TOPSAN pages increasingly do not focus on individual proteins, but on protein families, specific organisms, or pathways (**Figure **2). Since all information (including both positive and negative data) provided on the TOPSAN page is public, issues of data ownership, which typically limit access to unpublished data, do not impede analyses by third-party participants.

Annotation and analysis of every protein usually use two main types of content. The first type is well-defined, with standardized data, such as amino-acid sequence, molecular weight, statistical significance of its structural similarity to other proteins, and structural or functional classification. Such content is downloaded or linked from relational databases or third-party resources and/or precomputed by the automated annotation scripts. The second type, which heavily relies on the first, consists of interpretation of the results of the first type of content and the results of additional, specialized experiments and is typically provided by human experts. TOPSAN pages (see **Figure **2) provide both types of content: the first is from predefined windows displaying data parsed for databases and precomputed by annotation scripts, and the second is via fields that can be edited by a WYSIWYG editor and can contain figures prepared by a user.

The current TOPSAN implementation contains over 10,000 protein pages and covers all proteins whose structures were determined by various SG centers; pages for over 4,500 proteins targeted by the JCSG, but not yet determined (i.e., they are at various stages of protein production and structure determination), and several hundred user-created pages that do not fall into the above categories. Over 1,000 pages contain human-curated annotations, ranging from a few sentences to several pages, with a median of over 150 words, and of quality from relatively trivial annotations to publication-quality manuscripts. The content to date has been amassed from over 400 registered users, although the majority has been contributed by a smaller subset of the most active contributors. While most of the TOPSAN pages focus on experimentally determined protein structures, TOPSAN was of great utility in the development of the complete metabolic reconstruction for *Thermotoga maritima *[20], in which the annotations and the analysis focused on protein function and included many proteins for which only predicted models were available (since structures for over one-half of proteins in the reconstruction were not determined experimentally). TOPSAN is also an integral part of the structure determination pipeline at JCSG. This pipeline is both a physical (or an experimental) process involving the handling and processing of proteins and their crystals samples, and also a flow of information about the samples. This latter process starts from the initial automated annotations performed at the stage of target selection, continues with the collection of all experimental data on each sample, and concludes with the assembly of a full set of coordinates for deposition to PDB. In this context, TOPSAN pages, which initially contain only the automated annotations, are used as a notebook where JCSG scientists register their observations during the refinement of the structural model prior to its deposition in PDB. These initial comments are then expanded by discussion and idea exchange between JCSG scientists and/or other users of the system.

TOPSAN aims not only to collect information about proteins whose structures have been determined, but also to share and disseminate this information. TOPSAN pages are free to view without registration, and TOPSAN content is licensed under the Creative Commons share-alike license http://creativecommons.org/licenses/by-nc-sa/3.0/legalcode, which allows content to be available for others to build upon and share legally. In addition to open-content licensing, TOPSAN provides an application programming interface (API) that enables external sources to retrieve content easily. API access is available to collaborating sites to retrieve content from TOPSAN protein pages. This has been implemented for PFAM, where the contents of an individual TOPSAN protein page are currently being imported to PFAM by a PFAM server via the TOPSAN API. This feature is standard in the PFAM 24.0 release. In this case, we developed a custom PHP script (files.topsan.org/retrieve.php?uniprotId = xxx) for PFAM servers to readily access TOPSAN page content in real time. A structured XML page is provided for each protein on TOPSAN. A Uniprot ID is passed as an argument to the script, which then queries the TOPSAN MySQL database for the current annotation of the page as well as for any file attachments/images. This information is formatted as an XML document and returned to the requesting client. The API is flexible and can easily be used for third parties to retrieve our content. TOPSAN pages are searchable with all Internet search engines; our internal analysis shows that about 50% of TOPSAN traffic "arrives" from Google and other search engines, and that users directly view specific protein pages that contain keywords that are a subject of an individual search. At the same time, the API access to TOPSAN allows for integration into other resources. For example, the Calit2 Visualization Lab at UCSD displays TOPSAN protein annotations from within their structure gallery CAVE 3D wall display. This protein structure display is utilized in courses both at UCSD and at The Scripps Research Institute.

### Implementation

The current implementation of TOPSAN was developed using MindTouch, an enterprise open-source collaboration and integration platform, which provides access to tools and scripting that can be utilized to develop a customized, interactive website. Scripts and customized templates defining TOPSAN-specific protein pages integrate several sources and types of information; namely, existing annotation automatically parsed from established resources and user-created content that can be edited by all registered users. The MindTouch platform offers some core features, such as open-source code; user account handling, including advanced security features; and extensive backup and version tracking capabilities that make it an excellent platform choice on which to build TOPSAN. At the same time, MindTouch is comparable in many ways to other platforms. For instance, the initial version of TOPSAN, which was in use until late 2008, was developed using the now-defunct JOTSPOT platform; the TOPSAN server was then migrated to the MindTouch platform, maintaining its overall look, feel, and functionality.

**Figure 3:**
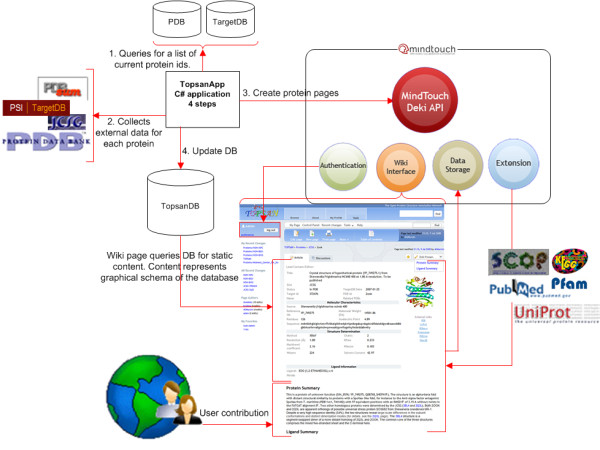
**TOPSAN is developed on the Mindtouch framework http://www.mindtouch.com/, which adheres to open-source standards and has an extensive set of features that can be readily modified for our exploratory platform**. The remote application TopsanApp uses a REST API to generate and automatically populate TOPSAN entries with content from a variety of primary and derived data sources. The wiki interface is used for community input.

A remote application (TopsanApp) is used to retrieve protein information from external resources and to create and store pages for specified proteins on the platform through an API. This application is coded in C# built on the .NET framework (Figure 3) and first collects a list of protein IDs (targetID) from TargetDB [21], which includes information about all protein targets of SG centers. Once a list of IDs has been constructed, a series of external data sources are queried to retrieve information for each protein. Information, such as sequence, contributing laboratory, PDB ID, source organism, TargetDB date of update, and status of the protein in the production pipeline, are obtained. From the sequence, the molecular weight and isoelectric point are calculated. The PDB is then queried for any experimental details, which are then stored in a mySQL table, pdbData. For proteins with a PDB ID, PDBSum [22] is queried for ligand information. Another Python script generates images for all protein structures by programmatically accessing the protein structure visualization program PyMOL and generates a customized view of the protein. All collected data are stored in a local MySQL database, topsanDB. The static content on a protein page is representative of the topsanDB schema. Next, a page is created on TOPSAN via MindTouch's REST [23] API, which has its own scripting language, dekiscript, with built-in functions for easily accessing and manipulating information. The content within each protein page contains dekiscript to query topsanDB to retrieve protein information. This information is passed as arguments to a template, which is embedded on each protein page and displays all the content in the format defined in the template. Templates allow for structure and uniformity across the pages and are an easy way to make changes quickly across all protein pages.

Access to view or edit a page can be set for all protein pages to be at the individual or group level. As part of our customization, we have developed a registration process that allows for some level of initiation and management of new users. All unregistered guests have access to view all protein pages on TOPSAN. Upon registration, a profile page, which may be edited by the new user, is created. It is here that they may enter scientific interests, upload profile images, and add their publications. A user's profile page is linked to every protein page contribution thereafter. After registration, the site administrator conducts a basic verification procedure that includes checking the user's e-mail address and cursory qualifications to ensure a level of validity of the user and protect the system from abuse and spam. Upon successful verification, a user is given "Contributor"-level permissions that allow him/her to edit all protein pages and respective discussion pages.

A new protein page can be created on TOPSAN when requested by a user, but can be also created automatically by the system. At this point, pages are created automatically for all JCSG targets when they pass the crystallization stage and for all targets from other PSI centers when structures are deposited in PDB. At any moment, two fields--protein summary and ligand summary--are available for editing to registered users. Other fields on the page, including links to precalculated analyses, are prepared automatically and placed there by annotation scripts based on parsing of input from several databases, such as targetDB or PDB, and annotation/analysis servers, such as PFAM [24], SCOP [25], PDBSum [22], FFAS [26], and others. Links to other annotation resources, such as Proteopedia [9], are also available, although it is expected that a majority of proteins annotated in TOPSAN would not have significant Proteopedia (or any other Wikipedia-like) coverage as, in most cases, no publication would be available and such annotation resources are restricted by the Wikipedia model. We expect, however, that as TOPSAN entries mature into complete publications, this knowledge will eventually feed into these encyclopedic systems.

### Beyond Individual Protein Structures

An important part of studying a protein is comparing it to other proteins. TOPSAN allows users to create groups of proteins using a tagging system. This feature gives the user the ability to create customized higher-order groupings based on their own criteria, expanding on the predefined classification systems already in use in the scientific community, which were mapped onto TOPSAN proteins using a similar system. For instance, SCOP hierarchical classification protein systems were mapped onto TOPSAN, where each protein that has a SCOP entry can be annotated at its respective Class, Fold, Superfamily, or Family level, as defined in SCOP. In addition to allowing users to describe features at the higher order of the individual structure, community-based structure classification to the SCOP hierarchy could be propagated back to SCOP. Utilizing pre-defined classification systems on TOPSAN to create groupings of proteins for higher-order annotations is currently being expanded to use CATH [27], PFAM [24], and metabolic pathways [20].

### TOPSAN-enabled collaborations

A TOPSAN page usually starts from a simple summary of the structure and (if known) functional annotations of the family to which it belongs. Very often, distant structural similarity provides additional hints, leading to further questions and hypotheses. For instance, the crystal structure of the protein NE1406 from *Nitrosomonas europaea*, solved by the JCSG in 2006 and deposited in PDB with code 2ich, was the first structure for the PFAM09410 protein family (unpublished observation). This family, called DUF2006 (Domain of Unknown Function #2006) has over 400 homologs in bacteria, archaea, and fungi. The PFAM DUF2006 family overlaps with the COG5621 family of predicted secreted hydrolases (COG5621) and is distantly related to two other PFAM families of hydroxyneurosporene synthases (PFAM0743) and Svf1-like proteins (PF08622). While this information was available prior to structure determination, structure analysis of NE1406 subsequently revealed that it consists of two repeats of a novel variant of an up-and-down β-barrel structure and is structurally similar to proteins from the calycin superfamily of integral transmembrane proteins in mitochondria and in the outer membrane of Gram-negative bacteria. This structural similarity, accompanied by very low sequence similarity (the structural alignment shows only a 3% sequence identity) led to an ongoing debate about possible homology and functional similarity between these two groups of proteins. On one hand, the characteristic sequence signature of the calycin superfamily was present in the N-terminal half of NE1406 (but absent from the C-terminal half) and, in addition, two (out of three) short conserved regions (SCR), characteristic of the lipocalin family, were also present in NE1406. On the other hand, analysis of the structural superposition of NE1406 with members of the calycin superfamily revealed a number of systematic differences; the β-sheets forming the NE1406 barrel were both longer and flatter than those in calycins, resulting in a narrower opening at the bottom of the barrel, at the site of the binding site in calycins. Secondary structure elements, such as the long C-terminal α-helix characteristic of lipocalins (e.g., nitrophorin), which represent a structurally and functionally distinct subclass [27] of the calycins, were also absent from NE1406. Finally, the signature residues were in different conformations, with Trp50 adopting a different rotamer in NE1406 than normally found in calycins and Arg214 adopting a more compact rather than fully extended conformation.

**Figure 4:**
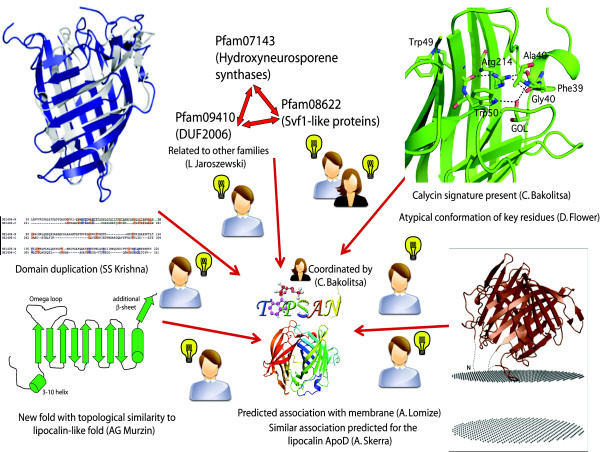
**Through TOPSAN collaboration, the first structural representative of a domain of unknown function was determined to be a lipocalin-related protein**. Individual collaborators contributed elements that would have remained at the "curiosity" level, but, collectively, the combined information led to a confident functional assignment resulting in a peer-reviewed publication.

Experimental verification of the NE1406 connection to calycins or lipocalins would present the first evidence of a lipocalin-related protein in the archaea domain and would settle the question of whether this family may have arisen via horizontal transfer to eukaryotic cells from the endosymbiotic alpha-proteobacterial ancestor of the mitochondrion [27]. This annotation was a collaborative effort that played out on TOPSAN, and a schematic history of this annotation is illustrated in **Figure **4, which represents the summary of the discussion on the TOPSAN page http://www.topsan.org/explore?pdbid=2ich and has contributions from Alexey Murzin, Arne Skerra, Andrei L. Lomize, Darren Flower, and members of the JCSG team. This type of interaction involving scientists in several locations around the globe could have happened naturally over a longer period of time using standard interaction mechanisms, such as chance encounters in scientific meetings. (Many such interactions have, of course, taken place in the history of science.) However, the TOPSAN platform allowed this interaction to happen in cyberspace over the course of a few days among participants who never met in person and some of whom did not even know each other.

## Discussion and Conclusions

In this article, we describe TOPSAN, The Open Protein Structure Annotation Network http://topsan.org, a collaborative environment developed by the Bioinformatics Core of the Joint Center for Structural Genomics to facilitate annotations and collaborative research in order to characterize protein structures solved by Protein Structure Initiative production centers and other structural genomics groups, and in turn, to facilitate the integration of these structures with research in the broad biological and biochemical community. Structural genomics, in its quest to provide broad coverage of protein space, frequently targets uncharacterized proteins, whose specific functions are unknown. Sometimes, an analysis of distant homology relationships or of the data from expression arrays suggest a possible relation to another, better-characterized protein family, but the reliability of such predictions and the extent of possible functional similarity varies from case to case and needs expert analysis. For other structures, features that are novel and impossible to predict from the sequence can be gleaned from the structure and hint at possible, previously unknown twists in the evolution of some members of otherwise, well-characterized protein families. In many such cases, structure analysis may lead to a new structural or functional hypothesis or an interesting speculation, which while very intriguing, would likely remain unpublished using standard criteria for peer-review publications. TOPSAN opens up a venue for such information to become available to a wider audience, increasing the chance that such partial information would be debated, discussed, and eventually combined with other supporting information from experimental work in another lab or, encouragingly, would prompt another researcher to perform a critical experiment to evaluate and test the function or the hypothesis. TOPSAN strives to maintain quality that approaches peer-reviewed publications by ensuring the contributions come from registered users, who during registration have to present credentials as to their expertise in biology, as well as by monitoring and evaluating contributions to identify, on one hand, spammers or abusers of the system, and on the other, expert users who are ready to assist in ensuring quality by providing oversight of other users. With a mechanism of tracking authorship of contributions, TOPSAN offers great possibility that disparate collaborators, often unknown to one other, can pool their information and resources to arrive at a body of significant new knowledge. With these goals, TOPSAN aims to occupy a niche different from that of the Wikipedia-type scientific wikis and that of databases or depository sites. Nevertheless, the primary goal of both types of resources is to provide easier access to already existing and validated information. At the same time, with its emphasis on ease of use and its lack of requirements for a minimal contribution size, TOPSAN is different from the peer-reviewed, standard scientific literature and allows more spontaneous, rapid communication and quick interaction.

The TOPSAN project evolved from an internal JCSG effort to annotate and more fully characterize the proteins that were determined in our center. This history is responsible for the current implementation being strongly focused on protein structure, but the TOPSAN concept can be readily generalized to any high-throughput project, such as PSI:Biology, to novel structures of other macromolecules determined by a structural biology lab, and more broadly, to any research domain where there is a need for a wider collaboration and a chance that critical pieces of information or expertise for any given project or research area are already known or available from members of the scientific community. The essence of the TOPSAN approach is to encourage new collaborations and explore the use of diverse, often disparate data to find new, integrative views on important biological problems. Therefore, while at this point TOPSAN is an experiment in the annotation and analysis of proteins targeted by structural genomics, it is also a model for collaborations in the potentially much larger and more complex research fields that are emerging in biology and other research disciplines.

## Authors' contributions

DW developed the TOPSAN website, including page scripting and wrote sections of the paper; SSK developed the core concepts of TOPSAN, planned and supervised the development of TOPSAN implementation, and wrote sections of the paper; CB developed sample TOPSAN pages and edited numerous specific pages, contributed to the discussions about the TOPSAN concept, and wrote sections of the paper; IAW helped interconnect the TOPSAN concept with the research of the JCSG and provided input to the shape and form of TOPSAN pages; AG helped develop the TOPSAN concept, supervised the TOPSAN project, and wrote the paper; JW helped develop the TOPSAN concept, supervised the TOPSAN project, and wrote the paper

All authors participated in discussions and revisions of the paper. All authors read and approved the manuscript.
